# Supplementary material for: Network-based modelling reveals cell-type enriched patterns of non-coding RNA regulation during human skeletal muscle remodelling
Source: bioRxiv. 2024 Oct 9:2024.08.11.606848. Preprint. [Version 2] doi: 10.1101/2024.08.11.606848 (PMC11482748; doi:10.1101/2024.08.11.606848)
Supplement: Supplement 2 [file NIHPP2024.08.11.606848v2-supplement-2.pdf]

bioRxiv preprint doi: https://doi.org/10.1101/2024.08.11.606848; this version posted October 9, 2024. The copyright holder for this preprint (which was not certified by peer review) is the author/funder, who has granted bioRxiv a license to display the preprint in perpetuity. It is made available under aCC-BY 4.0 International license.

SUPPLEMENTAL MATERIALS

Title: Network-based modelling reveals cell-type enriched patterns of non-coding RNA regulation during human skeletal muscle remodelling

Authors: Jonathan C. Mcleod [1], Changhyun Lim[1,2], Tanner Stokes[1], Jalil-Ahmad Sharif [3], Vagif Zeynalli [1], Lucas Wiens [1], Alysha C D'Souza [1], Lauren Colenso-Semple [1], James McKendry [1,4], Robert W. Morton [1], Cameron J. Mitchell [5], Sara Y. Oikawa [1], Claes Wahlestedt [6], J Paul Chapple [3], Chris McGlory [7], James A. Timmons [3,6*] and Stuart M. Phillips [1*]

Short-title: RNA profiling of ncRNA genes in skeletal muscle

One Sentence Summary: We used an optimised transcriptomic strategy to identify a set of ncRNA genes regulated during skeletal muscle hypertrophy in one hundred and forty-four people, with network modelling and spatial imaging providing biological context.

Key words: Transcriptome, Angiogenesis, Spatial, Muscle Growth, Exercise, Single-Cell, Immune Cell

Affiliations:

[1]Department of Kinesiology, McMaster University, Hamilton, Ontario, Canada.

[2] Population Health Sciences Institute, Faculty of Medical Sciences, Newcastle University, Newcastle upon Tyne, UK

[3] Faculty of Medicine and Dentistry, Queen Mary University London, London, UK.

[4] Faculty of Land and Food Systems, Food, Nutrition & Health, University of British Columbia, BC, Canada

[5] School of Kinesiology, University of British Columbia, BC, Canada

[6] University of Miami Miller School of Medicine, Miami, FL, USA

[7] School of Kinesiology and Health Studies, Queens University, Kingston, ON, Canada

* Joint senior authors

Correspondence to: Jonathan C. Mcleod, Jonathan.mcleod@queensu.ca

Table S1. Demographics of the five studies used for establishing ncRNA genes associated with skeletal muscle hypertrophy.

	Morton et al., 2019 [40]	Morton et al., 2016 [39]	Phillips et al., 2017 [41]	Mitchell et al., 2014 [42]	Stokes et al., 2020 [25]
Sample size, n	32	33	47	20	12
Age, years	22 ± 3 (19 – 28)	23 ± 3 (20 – 29)	38 ± 9 (21 – 51)	24 ± 3 (20 – 30)	21 ± 3 (18 – 29)
Gender, M/F	32/0	33/0	18/29	20/0	12/0
Body mass index, kg/m^2	25 ± 6 (18 – 38)	26 ± 9 (41)	32 ± 4 (26 – 43)	24 ± 4 (15 – 32)	24 ± 3 (20 – 31)
Measurement instrument	DXA	DXA	DXA	MRI	DXA
Limbs Involved in Measurement	1	2	1	1	1
Pre-training LLM, kg	10.3 ± 2.1 (7.2 – 14.6)	24.2 ± 3.0 (17.4 – 30.9)	5.8 ± 1.4 (3.2 – 8.6)	-	9.5 ± 1.6 (7.7 – 13.4)
Post-training LLM, kg	10.6 ± 2.1 (7.6 – 15.2)	25.0 ± 3.0 (19.9 – 31.2)	6.0 ± 1.4 (3.5 – 8.7)	-	9.9 ± 1.6 (8.1 – 13.4)
Pre-training QMV, cm^3	-	-	-	1862.0 ± 402.6 (1039.0 – 2822.0)	-
Post-training QMV, cm^3	-	-	-	1985.0 ± 417.6 (1278.0 – 3104.0)	-
dLLM, %	3.2 ± 3.1 (-3.7 – 9.1)	3.0 ± 5.0 (-5.0 – 19.0)	3.1 ± 3.7 (-4.5 – 11.0)	-	5.0 ± 4.3 (-1.2 – 14.0)
dQMV, %	-	-	-	7.1 ± 7.0 (-1.8 – 24.7)	-
R^2 dLLM (kg) vs Pre-training LLM (kg)	0.07	0.01	< 0.01	-	< 0.01
R^2 dQMV (cm^3) vs Pre-training QMV (cm^3)	-	-	-	< 0.01	-
R^2 dLLM (kg) vs Age (years)	0.26	< 0.01	< 0.01	-	0.04
R^2 dQMV (cm^3) vs Age (years)	-	-	-	0.01	-
R^2 dLLM (kg) vs Gender	-	-	0.04	-	-
LMR group, n	20	20	24	14	10
NMLR group, n	10	13	20	5	2

Age, body mass index, Pre-training LLM, Post-training LLM, dLLM, Pre-training QMV, Post-training QMV, and dQMV are displayed as mean ± SD (min - max). Sample size, Gender, LMR group, and NMLR group are displayed as counts. Abbreviations: DXA, dual X-ray absorptiometry; MRI, magnetic resonance imaging; dLLM, delta leg lean mass; QMV, quadriceps muscle volume; LMR, lean mass responders; NMLR, no measurable lean mass response.

bioRxiv preprint doi: https://doi.org/10.1101/2024.08.11.606848; this version posted October 9, 2024. The copyright holder for this preprint (which was not certified by peer review) is the author/funder, who has granted bioRxiv a license to display the preprint in perpetuity. It is made available under aCC-BY 4.0 International license.

bioRxiv preprint doi: https://doi.org/10.1101/2024.08.11.606848; this version posted October 9, 2024. The copyright holder for this preprint (which was not certified by peer review) is the author/funder, who has granted bioRxiv a license to display the preprint in perpetuity. It is made available under aCC-BY 4.0 International license.

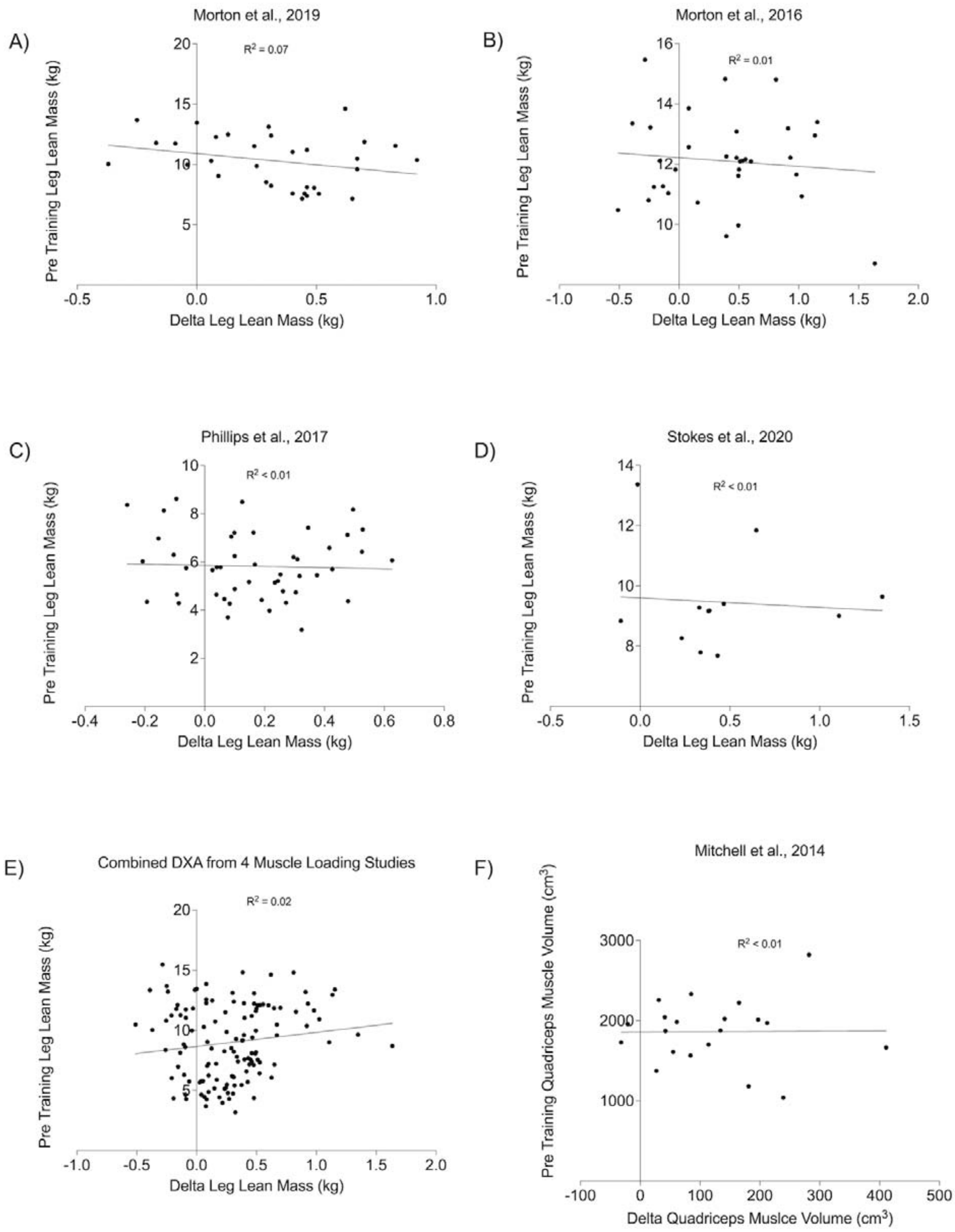

Figure S1. (A-D) Changes in leg lean mass versus pretraining leg lean mass for 4 muscle loading studies, and (E) depicts the aggregated relationship. F) Changes in quadriceps muscle volume vs pre-training quadriceps muscle volume.

bioRxiv preprint doi: https://doi.org/10.1101/2024.08.11.606848; this version posted October 9, 2024. The copyright holder for this preprint (which was not certified by peer review) is the author/funder, who has granted bioRxiv a license to display the preprint in perpetuity. It is made available under aCC-BY 4.0 International license.

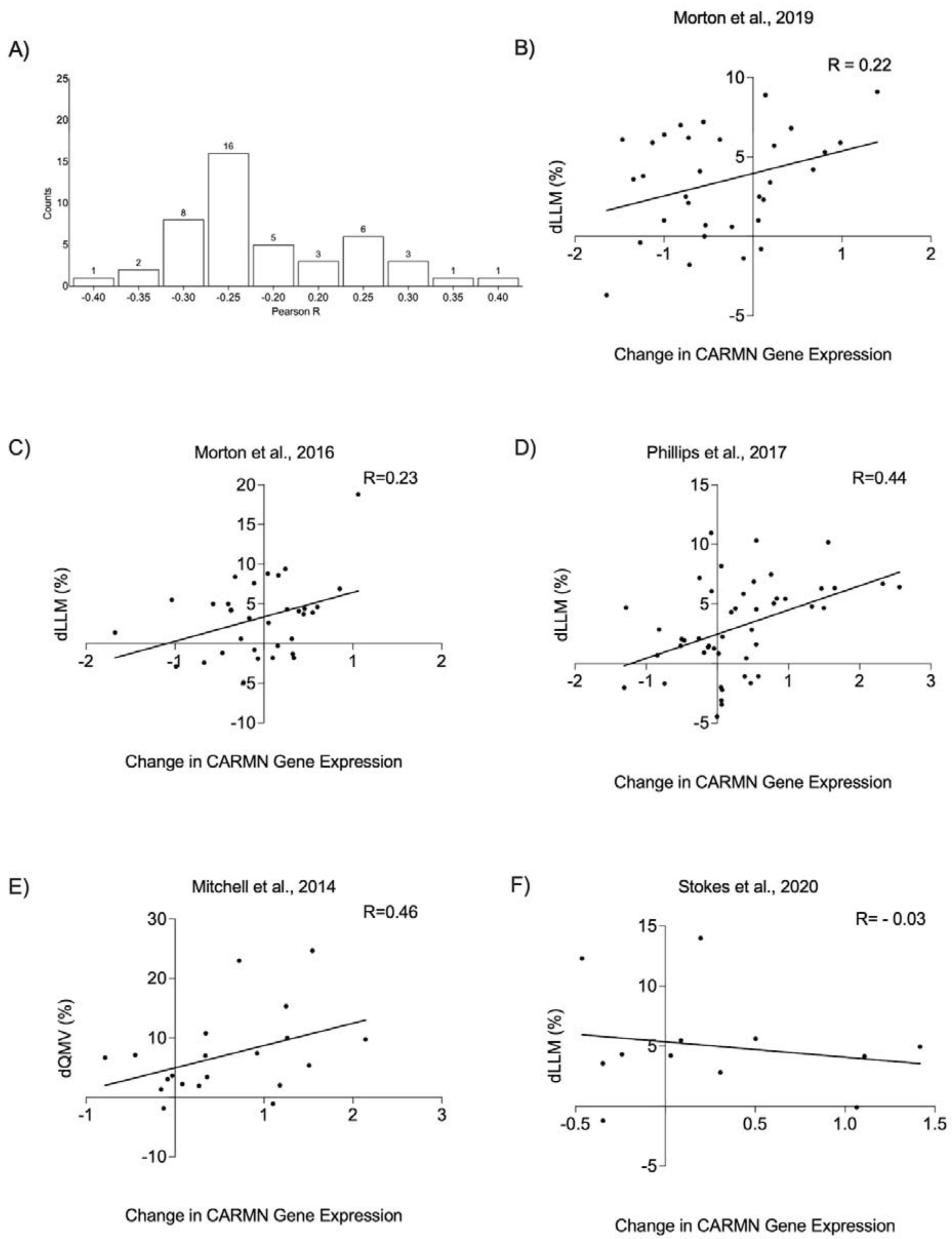

Figure S2. (A) Distribution of Pearson correlation coefficients among the 46 ncRNA genes containing a change in expression that was modestly associated with changes in LLM (dLLM), or changes in Quadriceps muscle volume (dQMV; Supplement Data S5). (B -F) Visual example

bioRxiv preprint doi: https://doi.org/10.1101/2024.08.11.606848; this version posted October 9, 2024. The copyright holder for this preprint (which was not certified by peer review) is the author/funder, who has granted bioRxiv a license to display the preprint in perpetuity. It is made available under aCC-BY 4.0 International license.

from the linear modelling analysis, depicting the relationship between dLLM (or dQMV [E]) and changes in *CARMN* gene expression for each of the 5 individual exercise studies.

bioRxiv preprint doi: https://doi.org/10.1101/2024.08.11.606848; this version posted October 9, 2024. The copyright holder for this preprint (which was not certified by peer review) is the author/funder, who has granted bioRxiv a license to display the preprint in perpetuity. It is made available under aCC-BY 4.0 International license.

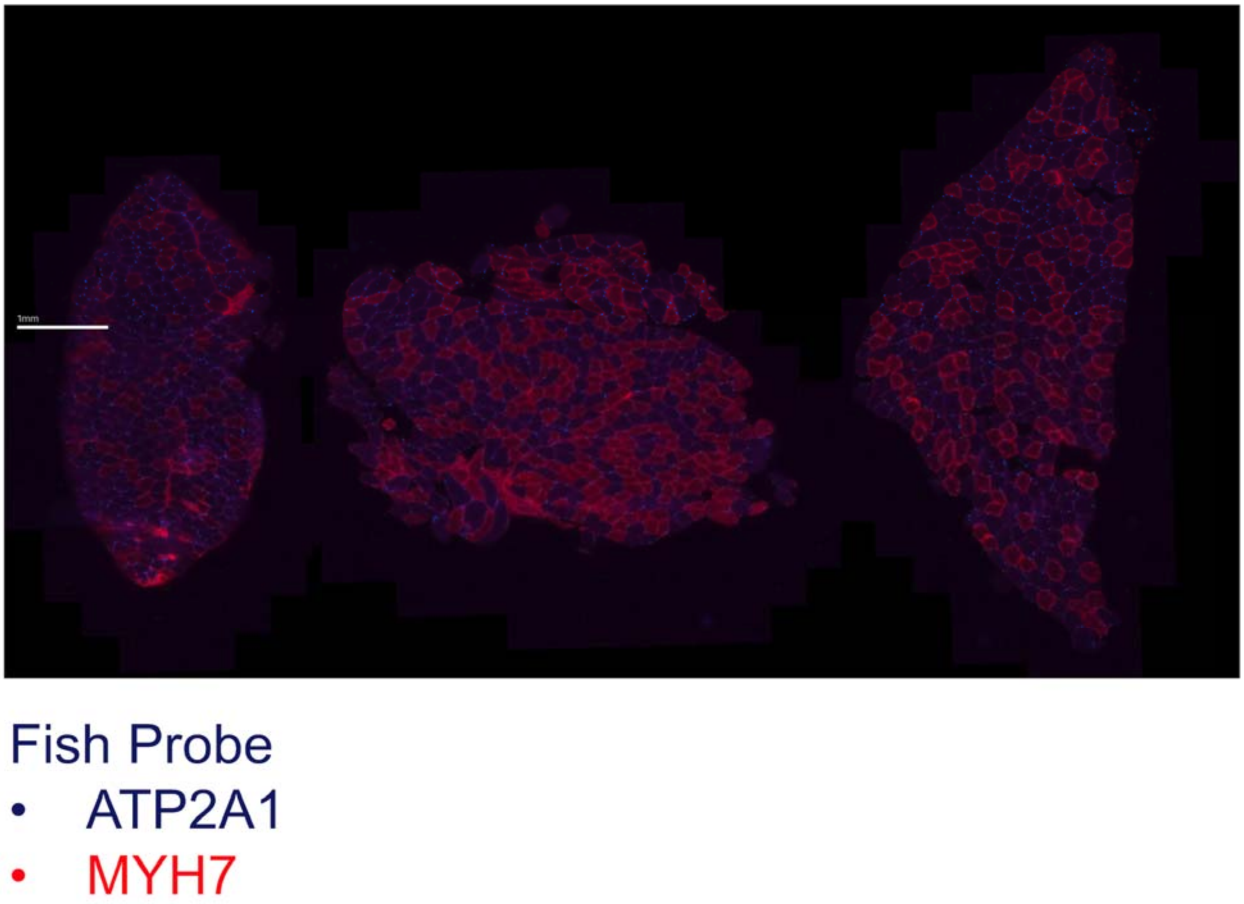

Figure S3. MERSCOPE-MERFISH overview for 3 samples used.

bioRxiv preprint doi: https://doi.org/10.1101/2024.08.11.606848; this version posted October 9, 2024. The copyright holder for this preprint (which was not certified by peer review) is the author/funder, who has granted bioRxiv a license to display the preprint in perpetuity. It is made available under aCC-BY 4.0 International license.

Table S2. Existing biochemical relationship for 110 hypertrophy-related ncRNA genes in skeletal muscle.

ENSG	Gene Symbol	Gene Synonyms	Group Identified In	Pre-existing evidence in skeletal muscle physiology
ENSG00000222041	CYTOR	C2ORF59, LINC00152, MGC4677, NCRNA00152	LMR	Exercise-induced lncRNA that is reduced with ageing. Promotes fast twitch cell fate. Reduces chromatin accessibility and sequesters TEAD1 [26].
ENSG00000268518	ENSG00000268518	MYREM, LNCFAM, LNCFAM71E1-2:2	LMR	Increases during myogenesis and promotes differentiation of myoblasts into myotubes [74]. Increases transcription of myosin binding protein C2 [74].
ENSG00000214548	MEG3	GTL2, LINC00023, NCRNA00023, ONCO-LNCRNA-83	LMR	MEG3 regulates myoblast differentiation [78]. MEG3 knockdown enhances mesenchymal characteristics, impairs myotube formation, and compromises skeletal muscle regeneration [78].
ENSG00000130600	H19	ASM, ASM1, D11S813E, LINC00008, MIR675HG, NCRNA00008	LMR	Predictor VO2max training response [77]. H19-encoded miRNA regulates skeletal muscle growth and translation initiation factors [99]. Maintains slow muscle fibre phenotype, exercise endurance and muscle function in mice [130]. In mice myoblasts, H19 associates with Dystrophin and protects its degradation [100].
ENSG00000241158	ADAMTS9-AS1	NONE	dLLM vs dGE	NONE
ENSG00000262943	ALOX12P2_ENST00000570921	NONE	LMR	NONE
ENSG00000215811	BTNL10P	BTN4, BTNL10, BUTR1	dLLM vs dGE	NONE
ENSG00000254614	CAPN1-AS1	NONE	LMR	NONE
ENSG00000249669	CARMN	CARMEN, MIR143HG	LMR & dLLM vs dGE	NONE
ENSG00000272168	CASC15	LINC00340, LNC-SOX4-1	dLLM vs dGE	NONE
ENSG00000229140	CCDC26	MGC27434, RAM	dLLM vs dGE	NONE
ENSG00000234898	CHEK2P3	NONE	LMR	NONE
ENSG00000229452	CPVL-AS1	NONE	dLLM vs dGE	NONE
ENSG00000226950	DANCR	AGU2, ANCR, KIAA0114, LNCRNA-ANCR, SNHG13	LMR	NONE
ENSG00000233143	DIRC3-AS1	NONE	LMR	NONE
ENSG00000232010	DNMT3L-AS1	NONE	LMR	NONE
ENSG00000223668	EEF1A1P24	NONE	LMR	NONE
ENSG00000149656	ENSG00000149656	NONE	NMLMR	NONE
ENSG00000176349	ENSG00000176349	NONE	dLLM vs dGE	NONE
ENSG00000213963	ENSG00000213963	NONE	NMLMR	NONE
ENSG00000214942	ENSG00000214942	NONE	LMR	NONE
ENSG00000228778	ENSG00000228778	NONE	dLLM vs dGE	NONE
ENSG00000229425	ENSG00000229425	NONE	LMR	NONE
ENSG00000230947	ENSG00000230947	NONE	dLLM vs dGE	NONE
ENSG00000231927	ENSG00000231927	NONE	dLLM vs dGE	NONE
ENSG00000234139	ENSG00000234139	NONE	dLLM vs dGE	NONE
ENSG00000234677	ENSG00000234677	NONE	dLLM vs dGE	NONE
ENSG00000236234	ENSG00000236234	NONE	dLLM vs dGE	NONE
ENSG00000236601	ENSG00000236601	NONE	LMR	NONE

bioRxiv preprint doi: https://doi.org/10.1101/2024.08.11.606848; this version posted October 9, 2024. The copyright holder for this preprint (which was not certified by peer review) is the author/funder, who has granted bioRxiv a license to display the preprint in perpetuity. It is made available under aCC-BY 4.0 International license.

ENSG00000237035	ENSG00000237035	NONE	dLLM vs dGE	NONE
ENSG00000238142	ENSG00000238142	NONE	NMLMR	NONE
ENSG00000249021	ENSG00000249021	NONE	LMR	NONE
ENSG00000250978	ENSG00000250978	NONE	LMR	NONE
ENSG00000251511	ENSG00000251511	NONE	dLLM vs dGE	NONE
ENSG00000252230	ENSG00000252230	NONE	NMLMR	NONE
ENSG00000253553	ENSG00000253553	NONE	dLLM vs dGE	NONE
ENSG00000254409	ENSG00000254409	NONE	NMLMR	NONE
ENSG00000254641	ENSG00000254641	NONE	LMR	NONE
ENSG00000254975	ENSG00000254975	NONE	dLLM vs dGE	NONE
ENSG00000255313	ENSG00000255313	NONE	dLLM vs dGE	NONE
ENSG00000255689	ENSG00000255689	NONE	LMR	NONE
ENSG00000258649	ENSG00000258649	NONE	dLLM vs dGE	NONE
ENSG00000258760	ENSG00000258760	NONE	NMLMR	NONE
ENSG00000260971	ENSG00000260971	NONE	NMLMR	NONE
ENSG00000261327	ENSG00000261327	NONE	dLLM vs dGE	NONE
ENSG00000263618	ENSG00000263618	NONE	LMR	NONE
ENSG00000267784	ENSG00000267784	NONE	LMR	NONE
ENSG00000273674	ENSG00000273674	NONE	dLLM vs dGE	NONE
ENSG00000279482	ENSG00000279482	NONE	LMR	NONE
ENSG00000285945	ENSG00000285945	NONE	LMR	NONE
ENSG00000286829	ENSG00000286829	NONE	LMR	NONE
ENSG00000288253	ENSG00000288253	NONE	dLLM vs dGE	NONE
ENSG00000289228	ENSG00000289228	NONE	LMR	NONE
ENSG00000290482	ENSG00000290482	NONE	dLLM vs dGE	NONE
ENSG00000291041	ENSG00000291041	NONE	LMR	NONE
ENSG00000273100	ENST00000610240	NONE	LMR	NONE
ENSG00000275563	ENST00000613990	NONE	LMR	NONE
ENSG00000260528	FAM157C	NONE	NMLMR	NONE
ENSG00000251402	FAM90A25P	NONE	LMR	NONE
ENSG00000256943	GALNT9-AS1	NONE	dLLM vs dGE	NONE
ENSG00000224934	GOT1-DT	NONE	LMR	NONE
ENSG00000179362	HMGN2P46	C15ORF21, D-PCA-2, FLJ39426	dLLM vs dGE	NONE
ENSG00000224699	LAMTOR5-AS1	NONE	dLLM vs dGE	NONE
ENSG00000230710	LINC00332	NCRNA00332	LMR	NONE
ENSG00000226519	LINC00390	TCONS_00021641	LMR	NONE
ENSG00000236678	LINC00347	NONE	LMR	NONE
ENSG00000225179	LINC00457	NONE	dLLM vs dGE	NONE
ENSG00000233723	LINC01122	AC007092.1, FLJ30838	dLLM vs dGE	NONE

bioRxiv preprint doi: https://doi.org/10.1101/2024.08.11.606848; this version posted October 9, 2024. The copyright holder for this preprint (which was not certified by peer review) is the author/funder, who has granted bioRxiv a license to display the preprint in perpetuity. It is made available under aCC-BY 4.0 International license.

ENSG00000233985	LINC01681	NONE	dLLM vs dGE	NONE
ENSG00000226983	LINC01692	NONE	dLLM vs dGE	NONE
ENSG00000237166	LINC01792	NONE	dLLM vs dGE	NONE
ENSG00000267057	LINC01905	NONE	LMR	NONE
ENSG00000204650	LINC02210	C17ORF69, C17orf69, CRHR1-IT1, FLJ25168	NMLMR	NONE
ENSG00000229536	LINC02572	AC079776.2	NMLMR	NONE
ENSG00000233593	LINC02609	NONE	dLLM vs dGE	NONE
ENSG00000234222	LIX1L-AS1	NONE	LMR	NONE
ENSG00000267023	LRRC37A16P	NONE	LMR	NONE
ENSG00000254349	MIR2052HG	NONE	NMLMR	NONE
ENSG00000172965	MIR4435-2HG	AGD2, AK001796, LINC00978, LNCRNA-AWPPH, MIR4435-1HG, MORRBID	LMR	NONE
ENSG00000197182	MIRLET7BHG	LINC-PPARA	dLLM vs dGE	NONE
ENSG00000238151	MLLT10P1	BA348I14.3, MLLT10L	LMR	NONE
ENSG00000242086	MUC20-OT1	NONE	LMR	NONE
ENSG00000214106	PAXIP1-AS2	PAXIP1OS	dLLM vs dGE	NONE
ENSG00000229941	PDE11A-AS1	NONE	dLLM vs dGE	NONE
ENSG00000249996	PPIC-AS1	NONE	dLLM vs dGE	NONE
ENSG00000226833	PPP1CB-DT	NONE	NMLMR	NONE
ENSG00000214182	PTMAP5	NONE	LMR	NONE
ENSG00000201875	RN7SKP178	NONE	LMR	NONE
ENSG00000275776	RN7SL185P	NONE	LMR	NONE
ENSG00000239468	RN7SL569P	NONE	dLLM vs dGE	NONE
ENSG00000239899	RN7SL674P	NONE	NMLMR	NONE
ENSG00000252957	RNA5SP402	NONE	dLLM vs dGE	NONE
ENSG00000201474	RNU6-164P	NONE	LMR	NONE
ENSG00000253084	RNU6-840P	NONE	LMR	NONE
ENSG00000199260	RNU6-874P	NONE	dLLM vs dGE	NONE
ENSG00000206732	RNU6-936P	NONE	dLLM vs dGE	NONE
ENSG00000230438	SERPINB9P1	MGC39372	LMR	NONE
ENSG00000234899	SOX9-AS1	NONE	LMR	NONE
ENSG00000187653	TMSB4XP8	TMSL3	LMR	NONE
ENSG00000232600	TONSL-AS1	NONE	dLLM vs dGE	NONE
ENSG00000237298	TTN-AS1	NONE	LMR	NONE
ENSG00000273249	WDR5-DT	NONE	NMLMR	NONE
ENSG00000199332	Y_RNA_ENST00000362462	NONE	NMLMR	NONE
ENSG00000199832	Y_RNA_ENST00000362962	NONE	LMR	NONE
ENSG00000212556	Y_RNA_ENST00000391254	NONE	dLLM vs dGE	NONE

bioRxiv preprint doi: https://doi.org/10.1101/2024.08.11.606848; this version posted October 9, 2024. The copyright holder for this preprint (which was not certified by peer review) is the author/funder, who has granted bioRxiv a license to display the preprint in perpetuity. It is made available under aCC-BY 4.0 International license.

ENSG00000252759	Y_RNA_ENST00000516950	NONE	dLLM vs dGE	NONE
ENSG00000252915	Y_RNA_ENST00000517106	NONE	dLLM vs dGE	NONE
ENSG00000250802	ZBED3-AS1	LNC13728	dLLM vs dGE	NONE
ENSG00000263072	ZNF213-AS1	NONE	dLLM vs dGE	NONE
ENSG00000257267	ZNF271P	HZF7, ZNF271, ZNFEB	LMR	NONE

A systematic PUBMED search was carried out on March 19th, 2024, using the gene symbol (and using any related gene synonyms found on Ensembl (https://useast.ensembl.org/index.html), and the following terms: “skeletal muscle” AND “myotubes” AND “myocytes”. Relevant articles were examined to determine a clear role in skeletal muscle physiology.

bioRxiv preprint doi: https://doi.org/10.1101/2024.08.11.606848; this version posted October 9, 2024. The copyright holder for this preprint (which was not certified by peer review) is the author/funder, who has granted bioRxiv a license to display the preprint in perpetuity. It is made available under aCC-BY 4.0 International license.

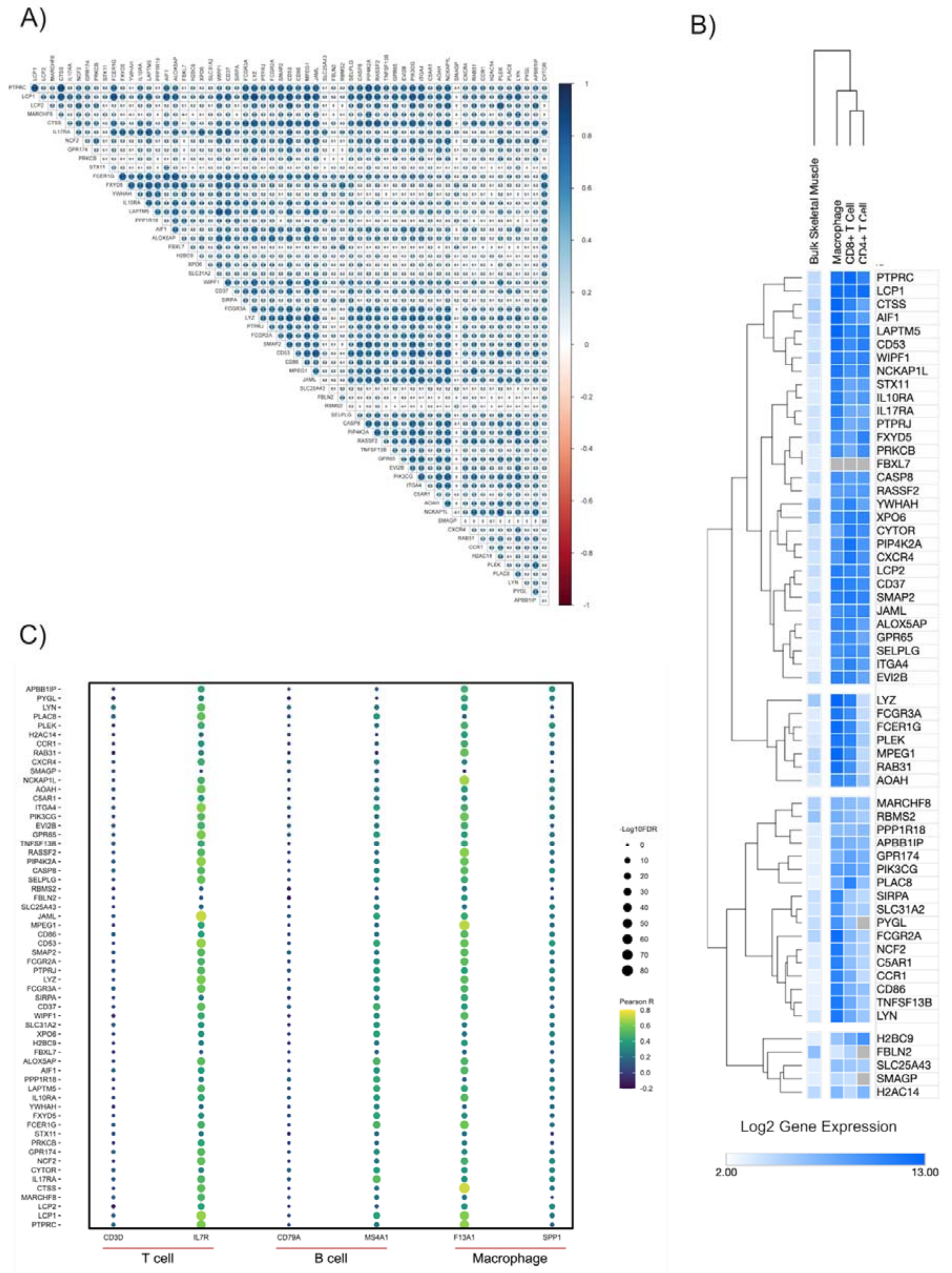

Figure S4. A) Pearson correlation matrix of all 60 genes co-expressed in network 1. Majority of the genes are positively correlated with each other in this network. B) the heatmap uses the log2 gene expression in skeletal muscle (n=437) and plots the 60 genes co-expressed in network 1

bioRxiv preprint doi: https://doi.org/10.1101/2024.08.11.606848; this version posted October 9, 2024. The copyright holder for this preprint (which was not certified by peer review) is the author/funder, who has granted bioRxiv a license to display the preprint in perpetuity. It is made available under aCC-BY 4.0 International license.

along with marker genes from three mononuclear cells of the immune system (macrophages, CD4 T-cells, and CD8 T-cells). The plot was created using Morpheus (https://clue.io/Morpheus), and genes and tissue types were hierarchically clustered using Euclidean distance (linkage method: complete). C) Dot plot depicting the association between the expression of network 1 genes, and gene markers for T-cells, B-cells and macrophages, in human skeletal muscle transcriptomic data (n=437). The colouring of the dot corresponds to the Pearson correlation coefficient, and the size of the dot is proportional to the –Log^{10} FDR.

bioRxiv preprint doi: https://doi.org/10.1101/2024.08.11.606848; this version posted October 9, 2024. The copyright holder for this preprint (which was not certified by peer review) is the author/funder, who has granted bioRxiv a license to display the preprint in perpetuity. It is made available under aCC-BY 4.0 International license.

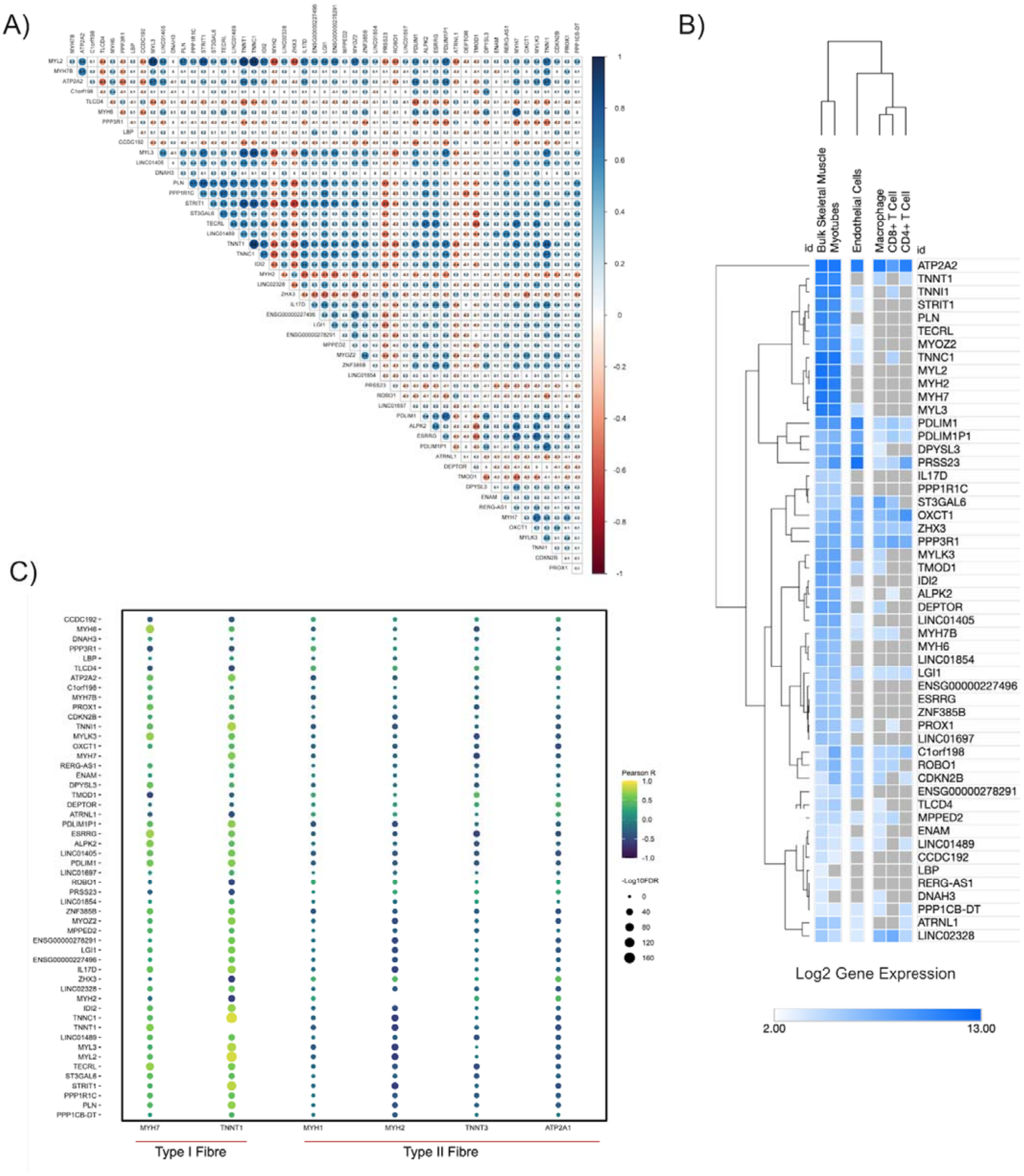

Figure S5. A) Pearson correlation matrix of all 52 genes co-expressed in network 2. Majority of the genes are positively correlated with each other in this network. b) the heatmap uses the log2

bioRxiv preprint doi: https://doi.org/10.1101/2024.08.11.606848; this version posted October 9, 2024. The copyright holder for this preprint (which was not certified by peer review) is the author/funder, who has granted bioRxiv a license to display the preprint in perpetuity. It is made available under aCC-BY 4.0 International license.

gene expression in skeletal muscle (n=437) and plots the 52 genes co-expressed in network 2 along with marker genes from bulk skeletal muscle, myotubes, endothelial cells, and three mononuclear cells of the immune system (macrophages, CD4 T-cells, and CD8 T-cells). The plot was created using Morpheus (https://clue.io/Morpheus), and genes and tissue types were hierarchically clustered using Euclidean distance (linkage method: complete). C) Dot plot depicting the association between the expression of network 2 genes, and gene markers for type I and type II fiber in human skeletal muscle transcriptomic data (n=437). The colouring of the dot corresponds to the Pearson correlation coefficient, and the size of the dot is proportional to the –Log^{10} FDR.

bioRxiv preprint doi: https://doi.org/10.1101/2024.08.11.606848; this version posted October 9, 2024. The copyright holder for this preprint (which was not certified by peer review) is the author/funder, who has granted bioRxiv a license to display the preprint in perpetuity. It is made available under aCC-BY 4.0 International license.

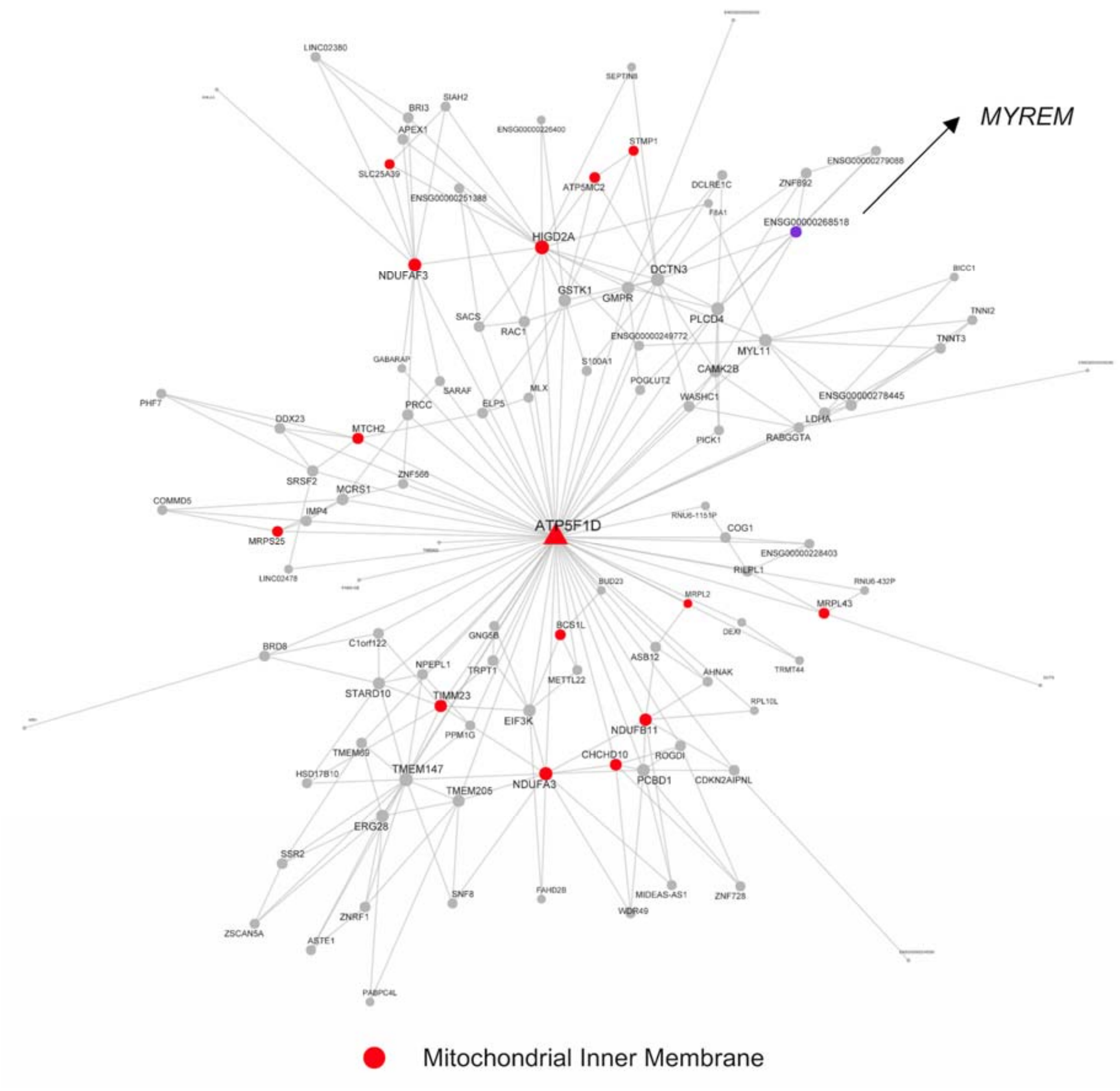

Figure S6. A mitochondrial-related gene co-expression network (network 4; Table 1) that contains the hypertrophy-related ncRNA gene, *MYREM* (purple).

bioRxiv preprint doi: https://doi.org/10.1101/2024.08.11.606848; this version posted October 9, 2024. The copyright holder for this preprint (which was not certified by peer review) is the author/funder, who has granted bioRxiv a license to display the preprint in perpetuity. It is made available under aCC-BY 4.0 International license.

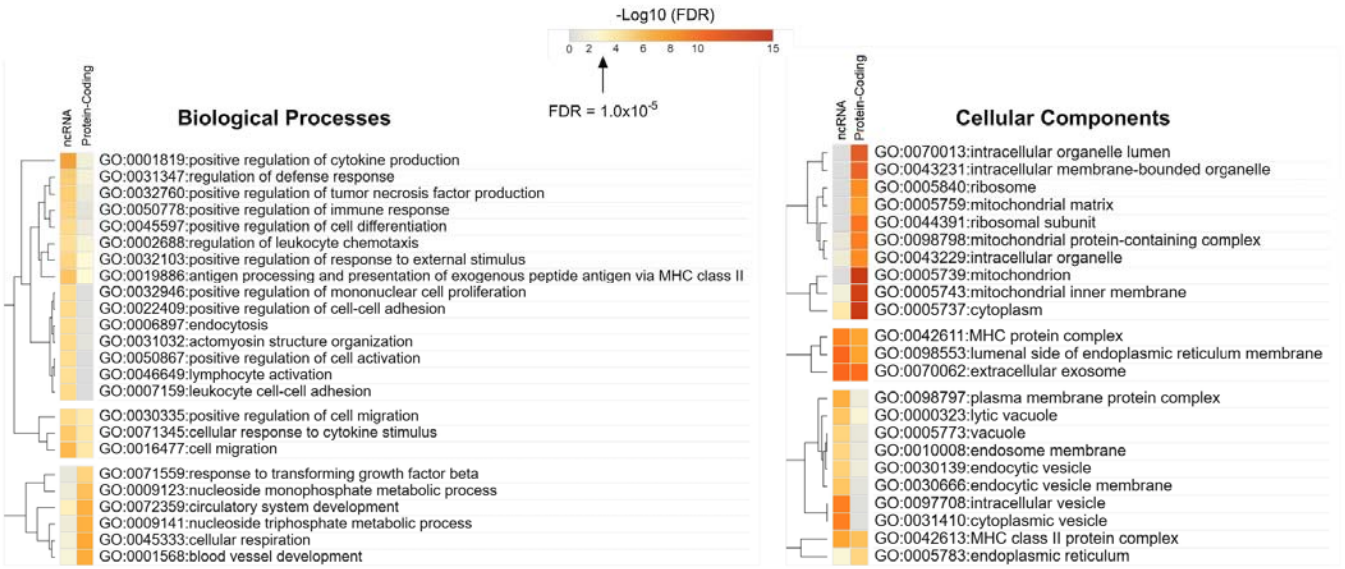

Figure S7. Similarities and differences in significant GO terms (biological processes and cellular components) across ncRNA MEGENA networks and our previously reported growth-regulated protein-coding MEGENA networks [25]. The heatplot was created using Morpheus (https://clue.io/Morpheus), and genes and tissue types were hierarchically clustered using Euclidean distance (linkage method: complete).
